# Self-esteem mediates the relationship between the parahippocampal gyrus and decisional procrastination at resting state

**DOI:** 10.3389/fnins.2024.1341142

**Published:** 2024-03-19

**Authors:** Weili Ling, Fan Yang, Taicheng Huang, Xueting Li

**Affiliations:** ^1^Department of Psychology, Renmin University of China, Beijing, China; ^2^Department of Psychology and Tsinghua Laboratory of Brain and Intelligence, Tsinghua University, Beijing, China

**Keywords:** decisional procrastination, the parahippocampal gyrus, self-esteem, individual difference approach, resting state

## Abstract

When faced with a conflict or dilemma, we tend to postpone or even avoid making a decision. This phenomenon is known as decisional procrastination. Here, we investigated the neural correlates of this phenomenon, in particular the parahippocampal gyrus (PHG) that has previously been identified in procrastination studies. In this study, we applied an individual difference approach to evaluate participants’ spontaneous neural activity in the PHG and their decisional procrastination levels, assessed outside the fMRI scanner. We discovered that the fractional amplitude of low-frequency fluctuations (fALFF) in the caudal PHG (cPHG) could predict participants’ level of decisional procrastination, as measured by the avoidant decision-making style. Importantly, participants’ self-esteem mediated the relationship between the cPHG and decisional procrastination, suggesting that individuals with higher levels of spontaneous activity in the cPHG are likely to have higher levels of self-esteem and thus be more likely to make decisions on time. In short, our study broadens the PHG’s known role in procrastination by demonstrating its link with decisional procrastination and the mediating influence of self-esteem, underscoring the need for further exploration of this mediation mechanism.

## 1 Introduction

Every day we encounter various situations that require decision-making, from food, clothing, and housing to jobs and marriage, and yet people have different habitual decision-making patterns (Basu and Dixit, 2022) that lead to significant individual differences in their decision-making styles (Li et al., 2017). For example, certain individuals tend to make decisions expeditiously, while others may delay or avoid decision-making altogether, particularly in situations involving conflict or dilemma. This latter propensity is termed decisional procrastination (Ferrari et al., 1995; Ferrari, 2000). Decisional procrastination is known to produce many aspects of negative outcomes, such as stress (Thunholm, 2008), burnout (Michailidis and Banks, 2016), anxiety (Pittig et al., 2015), and sleep disturbance (Salo and Allwood, 2011), and is therefore considered a risk factor for mental health (Bavolar and Bacikova-Sleskova, 2018). Previous studies have mainly focused on cognitive factors of decisional procrastination, such as the effects of task characteristics and environmental conditions (Devine and Kozlowski, 1995; Rusou et al., 2013; Berens and Funke, 2020), but the neural underpinnings of this phenomenon remain largely unexplored.

In contrast, extensive research has explored the neural correlates of general procrastination using various modalities such as structural, resting-state, and task-functional MRI (fMRI) (Zhang et al., 2016, 2021). Notably, the parahippocampal gyrus (PHG) has emerged as a pivotal region in general procrastination (Zhang et al., 2016, 2017; Liu and Feng, 2018; Chen et al., 2019; Gao et al., 2021). Voxel-Based Morphometry (VBM) analyses indicate that the gray matter volume of the PHG serves as a neural substrate for procrastination (Hu et al., 2018; Liu and Feng, 2018; Chen et al., 2019), with larger volumes correlating with increased procrastinatory behavior. Resting-state studies demonstrate a positive correlation between spontaneous neural activity in the PHG, quantified through z-score amplitude of low-frequency fluctuations (zALFF) and regional homogeneity (ReHo), and trait procrastination. It is suggested that heightened PHG activity, linked to processing negative future events, may contribute to elevated procrastination levels (Zhang et al., 2016). Functional connectivity studies show a nuanced relationship between the PHG and procrastination, including increased functional connectivity between the putamen and PHG associated with higher procrastination levels (Gao et al., 2021). Task-fMRI results also indicate abnormal PHG activity in individuals with alcoholism, who exhibit higher procrastination tendencies compared to the general population (Westgate et al., 2017).

Considering that decisional procrastination represents a specific facet of procrastination within the decision-making context (Gambetti et al., 2008; Gambetti and Giusberti, 2019), our study explores the potential role of the PHG in decisional procrastination. However, it is worth noting that the PHG comprises distinct functional subdivisions. Research suggests that its posterior segment is involved in processing visual–spatial information, while its anterior part is associated with contextual memory associations (Baumann and Mattingley, 2016) and the processing of emotional memories (Smith et al., 2004; Sterpenich et al., 2006). Furthermore, posterior PHG functions include mental simulation and episodic future thinking (Bastin et al., 2013; Bellmund et al., 2016). Notably, differences between decisional and general procrastination have been observed in various domains, including personality traits (Morris and Fritz, 2015), memory (Tibbett and Ferrari, 2015), and self-defeating behavior (Ferrari, 1994; Orellana-Damacela et al., 2000; Hen and Goroshit, 2020).

Given these distinctions, our study uniquely subdivides the PHG to investigate its distinct relationships with decisional procrastination. We employed resting-state fMRI to assess spontaneous neural activity in the PHG among a substantial participant cohort (*N* = 264). We examined the association between the fractional amplitude of low-frequency fluctuations (fALFF), a well-established indicator of local spontaneous neural activity (Zou et al., 2008; Wang et al., 2021), and decisional procrastination levels, as measured using the avoidant decision-making style subscale of the General Decision-Making Scale (GDMS) (Scott and Bruce, 1995). Notably, fALFF, a sensitive measure reflecting spontaneous neural activity, has been linked to local brain glucose metabolism (Aiello et al., 2015; Nugent et al., 2015; Marchitelli et al., 2018; Jiao et al., 2019). It quantifies the ratio of low-frequency power (0.01–0.08 Hz) to the entire frequency range (0–0.25 Hz). Importantly, fALFF is less susceptible to motion artifacts compared to functional connectivity (Yan et al., 2013) and exhibits improved sensitivity and specificity in detecting spontaneous brain activities (Zou et al., 2008; Zuo et al., 2010).

Having established a correlation between the PHG and decisional procrastination, our study delves further into the role of self-esteem in this relationship. This inquiry is informed by existing literature highlighting the substantial impact of self-esteem on decision-making styles, as proposed in the conflict theory of decision making (Mann et al., 1997). Evidence also supports PHG activation during self-esteem-related tasks (Onoda et al., 2010; Premkumar, 2012; Frewen et al., 2013). The conflict theory of decision-making posits that individuals’ concerns about potential decision errors leading to reputation and self-esteem loss can induce stress, impairing their ability to make high-quality decisions, such as opting for decisional procrastination (Mann et al., 1997). Consequently, lower self-esteem may lead to greater stress, defensive behaviors (Larrick, 1993), and higher levels of decisional procrastination. Indeed, self-esteem has been shown to be a critical predictor of general procrastination, with lower self-esteem individuals exhibiting higher procrastination levels (Ferrari, 1991). As decisional procrastination represents a specific facet of procrastination within decision-making (Gambetti et al., 2008; Gambetti and Giusberti, 2019), our study proposes that self-esteem, as a significant personality variable influencing procrastination, should similarly predict decisional procrastination levels. In summary, we suggest that the PHG’s impact on decisional procrastination may be mediated by self-esteem, considering self-esteem’s role in decision-making styles and its shared neural basis with procrastination in the PHG.

## 2 Methods

### 2.1 Participants

A total of 264 healthy college students (143 females; average age = 21.6 years; SD = 1.04) with no reported history of neurological or psychiatric disorders participated in this study. This research forms a part of the GEB project—an integrative investigation into the human mind, utilizing multimodal data on human behavior, neural anatomy, neural activity, and genetics (Huang et al., 2014; Song et al., 2014; Kong et al., 2015; Hao et al., 2016; Wang et al., 2018; Zhou et al., 2020; Yang et al., 2022). The study’s behavioral and fMRI protocols were authorized by the Institutional Review Board of Beijing Normal University. Before commencing the experiment, written consent was obtained from all participants.

### 2.2 Measurement on decisional procrastination

Decisional procrastination was measured using the avoidant decision-making style subscale of the General Decision-Making Scale (GDMS) (Scott and Bruce, 1995). This subscale comprises five items, including “I postpone decision-making whenever possible.” Participants rated their agreement with each item on a 6-point Likert scale, where 1 represents strong disagreement and 6 indicates strong agreement. Their total score, derived from all five items, reflected the extent of decisional procrastination, with a higher score indicating greater procrastination tendencies. The subscale showed satisfactory reliability in our sample, as evidenced by a Cronbach’s alpha of 0.85.

The General Procrastination Scale (GPS) (Lay, 1986) was also measured in order to examine the validity of the avoidant decision-making style subscale. The GPS includes 20 items, rated on a 6-point Likert scale from 1 (strongly disagree) to 6 (strongly agree). The cumulative score served as an indicator of participants’ general procrastination tendencies, with a higher score signifying greater inclination toward procrastination. The GPS demonstrated satisfactory reliability in our sample, as indicated by a Cronbach’s alpha of 0.91.

Self-esteem was evaluated using the Rosenberg Self-Esteem Scale (RSES) (Rosenberg, 1965), which includes 10 items rated on a 6-point Likert scale from 1 (strongly disagree) to 6 (strongly agree). An example item is “On the whole, I am satisfied with myself.” The total score was used to quantify participants’ self-esteem, with a higher score implying a more positive self-assessment of worth and value. In the present sample, the RSES demonstrated satisfactory reliability, with a Cronbach’s alpha of 0.89.

Given the documented association between intelligence and decision-making style (Di Fabio and Palazzeschi, 2012), we incorporated intelligence as a potential confounder in our analysis. We assessed participants’ intelligence using the Advanced Progressive Matrices (APM) test (Raven and Court, 1998). This test includes 36 items where participants choose the missing figure to complete a 3×3 matrix. The APM score, which is the count of items correctly answered within 30-min, represented each participant’s level of intelligence.

### 2.3 Resting state-fMRI data acquisition

We conducted the resting state fMRI (rs-fMRI) scan using a Siemens 3 T Magnetom Trio scanner, equipped with a 12-channel phased-array head coil, at the Beijing Normal University Imaging Center for Brain Research. For the 8-min scan, participants were instructed to stay awake, avoid systematic thought, keep still, and close their eyes. The scan comprised 240 contiguous echo-planar imaging (EPI) volumes with the following parameters: TR = 2000 ms, TE = 30 ms, flip angle = 90°, 33 slices, matrix = 64 × 64, FOV = 200 × 200 mm^2^, and acquisition voxel size = 3.1 × 3.1 × 3.6 mm^3^. For spatial registration, we collected high-resolution T1-weighted images using the magnetization prepared gradient echo sequence (MPRAGE: TR/TE/TI = 2530/3.39/1100 ms; flip angle = 7°; matrix = 256 × 256). We imaged 128 contiguous sagittal slices with an in-plane resolution of 1 × 1 mm^2^ and a slice thickness of 1.33 mm for full-brain coverage.

### 2.4 Rs-fMRI data preprocessing

We performed rs-fMRI preprocessing for each participant using FSL.^1^ The initial four volumes were discarded to allow for signal equilibrium. Further preprocessing steps encompassed spatial Gaussian smoothing (FWHM = 6 mm), realignment, head motion correction using MCFLIRT (aligning each volume to the image’s middle volume), mean-based intensity normalization, and linear trend removal. We registered each participant’s high-resolution anatomical image to the Montreal Neurological Institute 152-brain template (MNI152; 2 × 2 × 2 mm^3^ resolution) using a two-step process (Andersson et al., 2007). Initially, we employed FLIRT to perform a 12-degrees-of-freedom linear affine transformation. Then, we refined the registration using FNIRT nonlinear registration. To generate a six-degrees-of-freedom affine transformation matrix, we used FLIRT to register each participant’s functional images to the high-resolution anatomical images.

### 2.5 ROIs and mean fALFF values of ROIs

**Figure 1:**
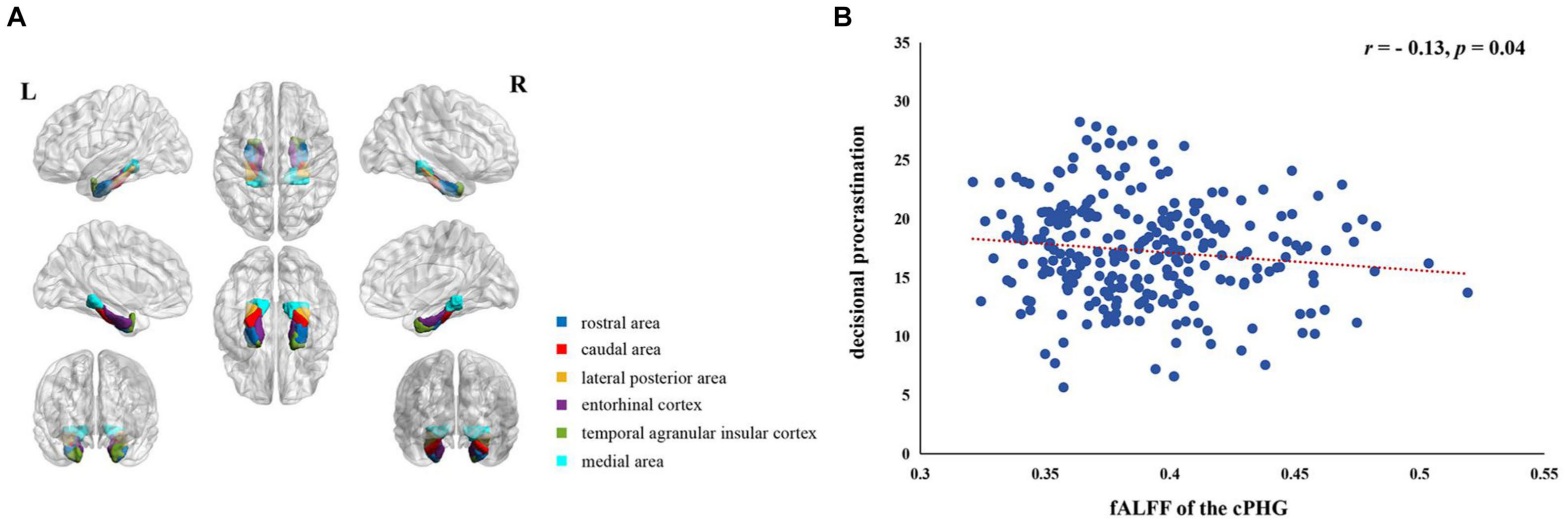
PHG subdivisions and neural correlates of decisional procrastination. **(A)** Each hemisphere’s PHG subdivisions are delineated in different colors, with the bilateral caudal area of the PHG depicted in red. **(B)** The scatterplot demonstrating the relationship between decisional procrastination levels and the mean fALFF values’ magnitude in the cPHG is presented for illustrative purposes.

Considering the PHG is a substantial brain region with multifaceted functions (Aminoff et al., 2007; Stenger et al., 2022), we segregated it into six subdivisions (Figure 1A) to explore the functions of each subdivision, respectively. These divisions, our regions of interest (ROIs), were based on the empirically validated Brainnetome Atlas (BNA) (Fan et al., 2016).^2^

We used SPM8 software^3^ within MATLAB R2019a (MathWorks Inc., Natick, MA, USA) to compute each ROI’s mean fALFF values. For each voxel’s time series, we obtained the sum of the amplitudes within the low-frequency range (0.01–0.1 Hz). The fALFF values for each voxel and each participant were then calculated by dividing this fractional low-frequency amplitude sum by the amplitude sum across the total frequency range (0–0.25 Hz) (Zou et al., 2008; Zuo et al., 2010). As a normalized ALFF index, fALFF minimizes the influence of artifactual signals near vessels or significant pulsatile motion (Zou et al., 2008; Zuo et al., 2010). The fALFF value maps were registered to the MNI152 space using the previously computed transformation matrix. Furthermore, we estimated each participant’s head motion from the rs-MRI data by incorporating the head motion correction outputs to further minimize the head motion’s influence.

### 2.6 fALFF-behavior correlation analysis

We implemented a partial correlation analysis to identify which PHG subdivision could predict decisional procrastination. For each of the six PHG ROIs, we examined the partial correlation between the mean fALFF values and avoidant decision-making style scores, with age, gender, intelligence (as measured by the APM), and head motion as control variables.

### 2.7 The mediation analysis

We performed a mediation analysis using the INDIRECT procedure with 5,000 bootstrap samples in SPSS (Preacher and Hayes, 2008) to investigate if individual differences in self-esteem could account for the relationship between the PHG’s fALFF values and decisional procrastination. In the mediation analysis, we studied the indirect effect of the fALFF values of the PHG (independent variable, IV) on avoidant decision-making style scores (dependent variable, DV) via self-esteem (mediator, M). We used a bootstrapping approach to test the indirect effect’s significance. The indirect effect estimate was derived from the mean of 5,000 bootstrap samples, with bias correction and acceleration accounting for 95% confidence intervals (Preacher and Hayes, 2008). The indirect effect was considered significant if the 95% confidence interval did not contain zero.

## 3 Results

Each participant’s decisional procrastination level was determined by their avoidant decision-making style score, with a higher score signifying a higher tendency to procrastinate or avoid decisions. The score’s kurtosis (−0.19) and skewness (0.05) suggest a normal distribution (Marcoulides and Hershberger, 2014) of decisional procrastination among participants. The mean was 17.33 and the SD was 4.41, indicating that the individual differences in decisional procrastination were large and thus suitable for exploring its neural correlates using the individual difference approach.

To validate the avoidant decision-making style as an accurate measure of decisional procrastination, we compared it against the General Procrastination Scale. A significant positive correlation was observed between decisional procrastination as measured by the avoidant decision-making style and general procrastination as measured by the GPS (*r* = 0.49, *p* < 0.001), which remained significant even after controlling for age, gender, and intelligence (*r* = 0.49, *p* < 0.001). Subsequently, we explored the relationship between the PHG and decisional procrastination.

In order to explore whether spontaneous activity in the PHG during resting state is associated with decisional procrastination, we examined the correlation between the mean fALFF values in the PHG and decisional procrastination levels. Our findings revealed a negative correlation (*r* = −0.11) between these two variables. However, this negative correlation did not reach statistical significance (*p* = 0.07). This outcome must be considered in the context that the PHG is a large brain area, comprising a total of 1767 voxels, with multiple subdivisions each performing different functions (Baumann and Mattingley, 2016) (refer to Methods, Figure 1A). Analyzing the PHG as a whole may potentially confound the effects of its distinct subregions on decisional procrastination, leading to biases in the results. Therefore, we examined the relationship for each of the 6 subdivisions separately. There was a significant inverse correlation between decisional procrastination levels and the mean fALFF values in the caudal area of PHG (cPHG, 312 voxels; MNI peak coordinates for left hemisphere x, y, z = −25, −25, −26; for right hemisphere x, y, z = 26, −23, −27) (*r* = −0.121, *p* = 0.049). This implies individuals with higher cPHG fALFF values were less prone to decisional procrastination. This correlation sustained even after controlling for age, gender, intelligence, and head motion (*r* = −0.126, *p* = 0.042). No significant correlations were detected in other PHG subdivisions (*p*s > 0.05) (Figure 1B).

Building upon the relationship between self-esteem, the PHG, and decisional procrastination, we examined if self-esteem mediates the correlation between the PHG and decisional procrastination. Echoing earlier findings (Mann et al., 1998; Thunholm, 2004; Deniz, 2006), a negative correlation was evident between self-esteem and decisional procrastination (*r* = −0.396, *p* < 0.001), indicating lower decisional procrastination in individuals with higher self-esteem. This correlation persisted when age, gender, and intelligence were controlled (*r* = −0.387, *p* < 0.001). Furthermore, there was a correlation between participants’ self-esteem levels and the fALFF magnitude in the cPHG (*r* = 0.13, *p* = 0.04), in line with previous research linking the PHG with self-esteem (Onoda et al., 2010; Premkumar, 2012; Frewen et al., 2013). Notably, after regressing for self-esteem variance, the correlation between the cPHG and decisional procrastination was not significant (*r* = −0.08, *p* = 0.18), suggesting that the relationship between the cPHG and decisional procrastination is reliant on self-esteem.

**Figure 2:**
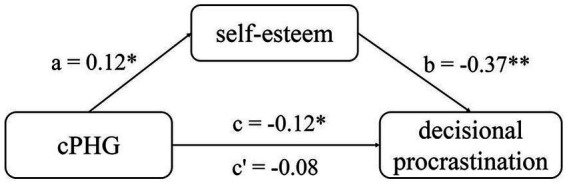
Mediation of the cPHG and decisional procrastination relationship by self-esteem path coefficients are displayed next to the arrows, indicating each link in the analysis. For the correlation between the cPHG and decisional procrastination, the lower value indicates the correlation after accounting for self-esteem as a mediator, whereas the upper value represents the correlation without adjusting for self-esteem’s effect. All values are standardized betas. * *p* < 0.05, ** *p* < 0.01, two-tailed.

To substantiate our hypothesis, we performed a mediation analysis, with the path diagram illustrated in Figure 2. Bootstrap simulation (*n* = 5,000) revealed a significant indirect effect via self-esteem (95% percentile CI = [−12.06 to −0.47], *p* < 0.05). This indirect effect remained significant even after controlling for age, gender, intelligence, and head motion (95% percentile CI = [−11.64 to −0.62], *p* < 0.05, see Figure 2), suggesting that the cPHG affects decisional procrastination through self-esteem. It’s worth noting that the direct effect in the mediation model was not significant (95% percentile CI = [−22.56 to 4.14], *p* > 0.05), implying that the correlation between the cPHG and decisional procrastination (*r* = −0.12, *p* = 0.04) lost its significance after adjusting for self-esteem (*r* = −0.08, *p* = 0.18). Hence, self-esteem fully mediates the effect of the cPHG on decisional procrastination.

## 4 Discussion

In this study, we utilized rs-fMRI to explore the neural underpinnings of decisional procrastination. We discovered that spontaneous neural activity within the caudal region of the PHG, as indicated by local fALFF, can predict decisional procrastination as measured by the avoidant decision-making style scale. This discovery broadens the PHG’s known role in general procrastination to the specific realm of decision-making. Moreover, our mediation analysis indicates that self-esteem serves as a bridge between the cPHG and decisional procrastination, suggesting that self-esteem could be a critical factor in how the PHG influences procrastination.

Our study is consistent with previous studies that the PHG is involved in procrastination (Zhang et al., 2016, 2017; Liu and Feng, 2018; Chen et al., 2019; Gao et al., 2021). Furthermore, our study extended the association between the PHG and general procrastination to procrastination in the domain of decision-making domain. This is important, because decision procrastination is considered different from general procrastination in many aspects. First, general procrastination is usually defined as a person’s voluntary postponement of an intended action despite the foreseeable negative consequences (Ferrari, 2010), whereas decisional procrastination indicates a strong tendency to fail to make timely decisions (Fabio, 2006) especially in stressful situations (Ferrari, 1994). That is, decisions are logically and psychologically preceded by action, and only when decisions have been made do individuals take action (Milgram and Tenne, 2000). Thus, decisional procrastination focuses on the early planning stages of the action process, such as information integration (Ferrari, 2000), whereas general procrastination is closer to action (Hen and Goroshit, 2020) and focuses on the behavioral component of procrastination. In addition, differences in decisional procrastination and general procrastination (Orellana-Damacela et al., 2000; Hen and Goroshit, 2020) are also found in personality traits (Morris and Fritz, 2015), memory (Tibbett and Ferrari, 2015), and the self-defeating behavior (Ferrari, 1994). Taken together, this distinction may explain that decisional procrastination was found to be associated with the caudal area of the PHG, whereas the neural correlates of general procrastination are identified in other subdivisions of the PHG (Zhang et al., 2016; Chen et al., 2019; Gao et al., 2021).

Unlike the anterior part of the PHG, the posterior part of the PHG that consists of the cPHG can be activated by episodic future thinking (Stawarczyk and D’Argembeau, 2015), which constructs a detailed, sensory-rich representation of future experiences and outcomes for decision-making based on the expectations of future events. Indeed, previous studies have found that the pessimistic and negative future thinking likely leads to decisional procrastination (Rebetez et al., 2016; Chen et al., 2019). Further studies have shown that individuals with lower self-esteem tend to have more negative future thinking and feelings of uncertainty about their abilities and future outcomes (Coruh and Vural, 2019; Ogasawara et al., 2020). Indeed, we found that self-esteem mediated the relationship between the cPHG and decisional procrastination. That is, the cPHG may be related to decision procrastination through the proxy of self-esteem.

Both the conflict theory (Mann et al., 1997) and the terror management theory (TMT) (Greenberg et al., 1986) consider self-esteem to be a central psychological factor influencing various human behaviors (Pyszczynski et al., 2004). And a large body of research has shown that self-esteem has a broad and profound effect on both procrastination and decision-making (Solomon and Rothblum, 1984; Ferrari, 1994; Beck et al., 2000; Ferrari, 2000; Deniz, 2006; Anthony et al., 2007; Farčić et al., 2020). Therefore, the mediating role of self-esteem in the relationship between the PHG and decisional procrastination may be enabled by its role in the domains of procrastination and decision-making. In the domain of procrastination, individuals with low self-esteem are more likely to procrastinate (Ferrari, 1991), possibly because they are concerned about their poor performance (Thompson, 2018). As a result, they avoid taking actions such as making decisions (Ferrari et al., 1995; Duru and Balkis, 2014) to avoid experiencing failure and to protect their self-worth. In the domain of decision-making, individuals with lower self-esteem perform worse in the decision-making, as they are more irrational (Mann et al., 1998; Thunholm, 2004; Deniz, 2006; Kumar, 2018), less confident in their decision-making abilities (Ogasawara et al., 2020) for good outcomes (Coruh and Vural, 2019). Unfortunately, the correlation analysis nature of this study cannot disentangle whether self-esteem mediates the relationship between the PHG and decisional procrastination in the domain of procrastination, or in the domain of decision-making, or both. Future research with causal designs may help to illustrate the mediating role of self-esteem in the link between the PHG and decisional procrastination.

While our study provides insightful observations regarding the relationship between the fALFF in the PHG and its 6 subdivisions with decisional procrastination, several limitations warrant consideration. The effect size observed in our research is relatively small. This could be attributed to our focused approach, which exclusively examined the PHG region. It is crucial to acknowledge that decisional procrastination, as a complex cognitive process, is likely influenced by a multitude of brain regions. Our study’s scope, limited to the PHG, may not capture the entire spectrum of neural correlates contributing to decisional procrastination. Moreover, we acknowledge the absence of multiple comparison corrections in our analysis of the relationship between fALFF in the PHG’s 6 subdivisions and decisional procrastination. Although we attempted to apply corrections using methods such as False Discovery Rate (FDR), Bonferroni, or Westfall-Young, our results did not withstand these adjustments. While such corrections are crucial for reducing false positives, they also carry the risk of introducing false negatives, potentially affecting the interpretation of our findings. It is important to contextualize our results in light of existing research, particularly studies focusing on the PHG and its relationship with procrastination (Zhang et al., 2016, 2017; Hu et al., 2018; Liu and Feng, 2018; Chen et al., 2019; Gao et al., 2021). Our findings, while limited, do align with these previous studies, suggesting a degree of consistency in the observed neural patterns related to decisional procrastination. In conclusion, while our study offers valuable insights into the neural basis of decisional procrastination, especially in relation to the PHG region, these limitations highlight the need for more comprehensive and multi-faceted research in this area. Future studies exploring a broader range of brain regions and incorporating robust statistical methodologies are essential to deepen our understanding of the neural mechanisms underpinning decisional procrastination.

## Data availability statement

The raw data supporting the conclusions of this article will be made available by the authors, without undue reservation.

## Ethics statement

The studies involving humans were approved by the Institutional Review Board of Beijing Normal University. The studies were conducted in accordance with the local legislation and institutional requirements. The participants provided their written informed consent to participate in this study.

## Author contributions

WL: Writing – original draft, Data curation, Formal analysis, Investigation, Project administration, Validation, Visualization. FY: Writing – original draft, Data curation, Formal analysis, Validation. TH: Writing – original draft, Resources, Software. XL: Writing – review & editing, Data curation, Formal analysis, Investigation, Project administration, Resources, Supervision, Validation, Visualization.

## Footnotes

^1^ www.fmrib.ox.ac.uk/fsl/

^2^ http://atlas.brainnetome.org

^3^ http://www.fil.ion.ucl.ac.uk/spm
